# Women’s psychological experiences of preterm labour and birth which results in an intrapartum stillbirth or a neonatal death: an empty systematic review

**DOI:** 10.3389/fpsyt.2025.1544485

**Published:** 2025-06-05

**Authors:** Semra Worrall, Elana Payne, Rebecca E. Fellows, Olivia Pike, Naomi H. Carlisle, Jenny Carter, Anja Wittkowski, Karen Burgess, Claire Storey, Laura A. Magee, Peter von Dadelszen, Paul Christiansen, Victoria Fallon, Asma Khalil, Sergio A. Silverio

**Affiliations:** ^1^ Department of Psychology, Institute of Population Health, University of Liverpool, Liverpool, United Kingdom; ^2^ Department of Women & Children’s Health, School of Life Course & Population Sciences, King’s College London, London, United Kingdom; ^3^ Department of Psychological Medicine, Institute of Psychiatry, Psychology & Neuroscience, King’s College London, London, United Kingdom; ^4^ Division of Methodologies, Florence Nightingale Faculty of Nursing, Midwifery & Palliative Care, King’s College London, London, United Kingdom; ^5^ School of Health Sciences, College of Medicine and Health, Bangor University, Bangor, United Kingdom; ^6^ Division of Psychology and Mental Health, School of Health Sciences, The University of Manchester, Manchester, United Kingdom; ^7^ PETALS: The Baby Loss Counselling Charity, Cambridge, United Kingdom; ^8^ Patient and Participant Involvement and Engagement Group for Perinatal Bereavement, Trauma, and Loss, King’s College London, London, United Kingdom; ^9^ Fetal Medicine Unit, Liverpool Women’s NHS Foundation Trust, Liverpool, United Kingdom; ^10^ Fetal Medicine Unit, St. George’s University Hospitals NHS Foundation Trust, University of London, London, United Kingdom

**Keywords:** preterm birth, neonatal death, intrapartum stillbirth, gestational age, perinatal mental health, empty systematic review

## Abstract

**Introduction:**

Nearly three quarters of stillbirths and neonatal deaths occur in infants born prematurely. The mothers of these children may be at increased risk of developing mental health difficulties as a result of their premature labour and/or subsequent loss.

**Methods:**

This systematic review was conducted to understand the psychological experiences of mothers who gave birth prematurely to a baby who subsequently dies as a result of an intrapartum stillbirth or a neonatal death. Ten databases were searched. Any studies which included women who had suffered a perinatal bereavement as a result of preterm labour and birth, in any country, and in any language were eligible to be included. Studies focusing on antepartum stillbirth or *in utero* death were excluded due to not having the element of preterm labour and/or birth within the studies. Risk of bias was to be assessed using the Critical Appraisal Skills Programme.

**Results:**

Following the screening of citations, no studies were eligible for inclusion in the review. The majority of studies were excluded due to a lack of distinction in terms of intrapartum or antepartum stillbirth, or grouping of types of perinatal loss. Had the inclusion criteria been less stringent and the three most common reasons for exclusion been removed, 19 studies would have been eligible for inclusion in the review, and we present a brief summary of these findings.

**Discussion:**

These review findings highlight the need for more research into the psychological experiences of mothers of preterm infants whose baby subsequently dies, whereby future studies should consider routine reporting of gestational age. To address the identified gaps, future research should consider alternative methods or broader inclusion criteria to capture relevant data. Emphasising the importance of reporting gestational age and distinguishing between types of perinatal loss will enhance the specificity of research findings.

## 1 Introduction

Preterm birth and perinatal bereavement continue to be neglected public health issues. Birth at <37 weeks’ gestation (‘preterm birth’) (1) is one of the leading causes of mortality worldwide in children under five (2). In the United Kingdom, prematurity is the leading cause of neonatal death (death of a live born infant within the first 28 completed days of life) (3), accounting for 73% of all infant mortality in 2021 (4). Although rates have been declining, predominantly due to increased medical advances in neonatal care, the downward trajectory is not stable, showing recent rises once again in 2021 (4).

The birth of a preterm baby can be psychologically traumatic for mothers. There is a well-documented increased risk of developing depression (5), anxiety (6, 7), and post-traumatic stress disorder (8), amongst mothers of premature infants, compared with those who deliver at term. Mental distress may be exacerbated further in women who give birth to extremely premature infants (e.g., <28 weeks’ gestation), due to increased health concerns associated with earlier gestational age (9). Mothers of premature infants commonly experience thanatophobic (extreme fear of death or the dying process) anxieties (10). Furthermore, the unexpectedness of the birth often renders mothers feeling unprepared for parenthood (11), whilst they face financial burdens and social isolation associated with the baby’s prematurity, which can persist well after birth (12). Intangible costs resulting from stillbirth such as grief and anxiety, as well as those more tangible such as financial difficulties, can be severe and long-lasting (13). These experiences may be exacerbated amongst those women whose infants require extended periods of time in neonatal care (7).

Prematurity remains the leading cause of stillbirth and neonatal death. Recent estimates suggest ≈75% of stillbirths and ≈73% of neonatal deaths occur in babies born prematurely (4); yet despite high prevalence rates, the psychological consequences of perinatal bereavement remain under-explored and under-reported (12). When the infant has a prolonged stay in hospital and their death is expected, mothers may experience feelings of fear and anticipatory grief (14). These psycho-emotional responses can often be difficult to communicate, because women simultaneously grieve the loss of their ‘normal’ pregnancy, alongside fearing for their infant’s life, and/or grieving following the death of their baby (13).

Despite the severe and long-lasting psychological consequences of both preterm birth and perinatal bereavement, there is no synthesis of the evidence outlining the psychological consequences for mothers who labour prematurely and whose preterm infant subsequently dies. Given that a review in this area had never been conducted, we kept the aim broad in scope. Thus, the current review aimed to understand the psychological experiences of women who went into preterm labour whose babies subsequently died due to an intrapartum stillbirth or a neonatal death, through a systematic review of both qualitative and quantitative literature.

## 2 Methods

### 2.1 Registration

**Figure 1:**
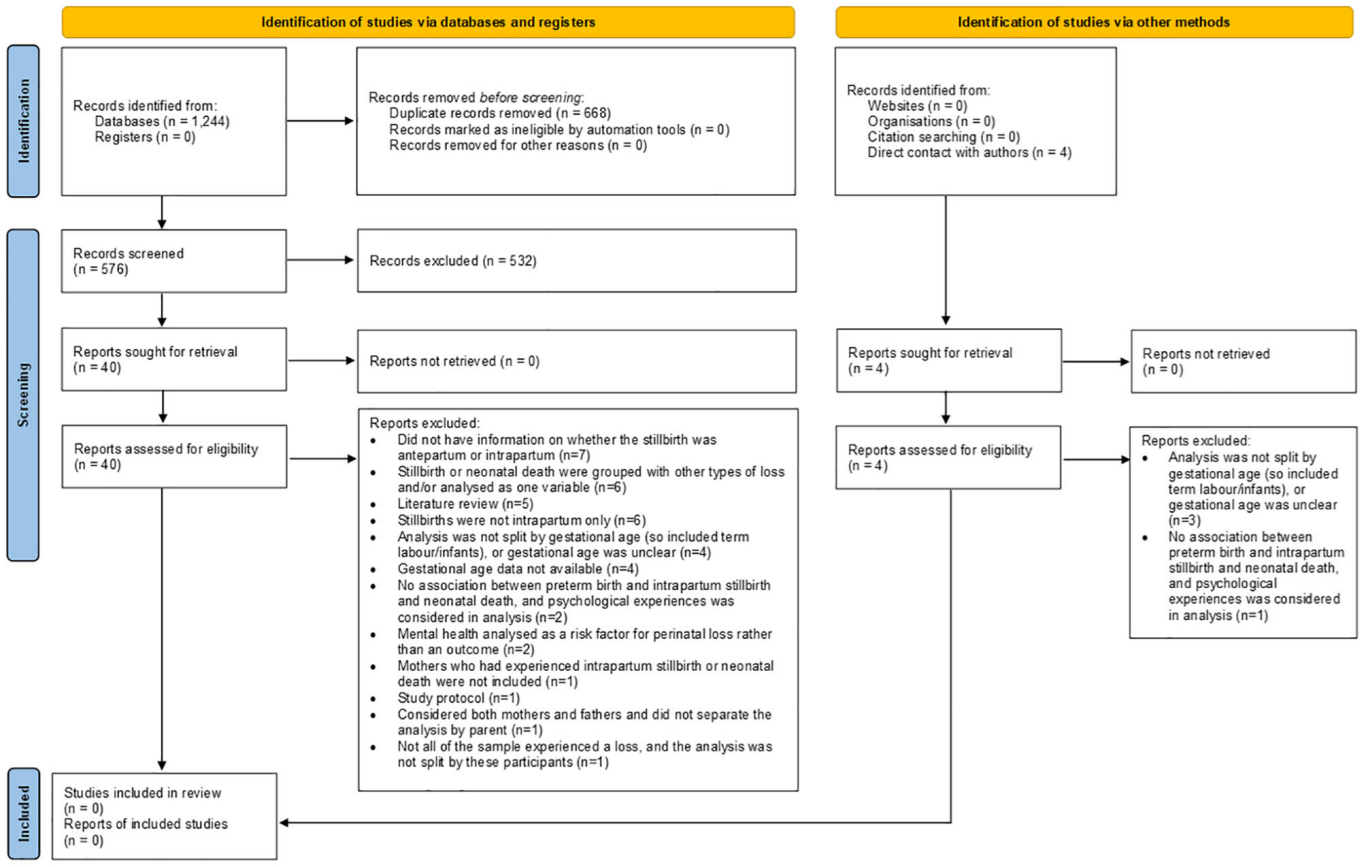
PRISMA 2020 flow diagram for new systematic reviews which included searches of databases, registers and other sources.

This systematic review was registered with PROSPERO in April 2024 (CRD42024516271) (15), and is reported according to Preferred Reporting Items for Systematic Reviews and Meta-Analyses [PRISMA] 2020 guidelines (16) (see **Figure 1**).

### 2.2 Patient and public involvement and engagement

The protocol for this systematic review was outlined and discussed at the Patient and Participant Involvement and Engagement [PPIE] Group for Perinatal Bereavement, Trauma, and Loss at King’s College London (18 March 2024). This group comprises experts by experience, members of third sector and charitable organisations, academics, researchers, clinicians, and policy makers. Through this engagement we sought and received feedback on the aim of the review from both lay and expert stakeholders, including members of the public, those with lived experience, health and social care professionals, researchers, and policy makers. They fully supported the rationale for the review and suggested we should augment the aim to also include stillbirth, as well as the originally intended inclusion of neonatal death in the review. These changes were incorporated before the PROSPERO registration.

### 2.3 Eligibility criteria

Studies were to be included if they reported a sample of women who laboured prematurely (up to 36^+6^ weeks’ gestation) and whose baby had subsequently died as a result of either an intrapartum stillbirth (a baby delivered at or after 24 completed weeks’ gestational age showing no signs of life and known to have been alive at the onset of care in labour) or a neonatal death (including early and late neonatal death) according to Mothers and Babies: Reducing Risk through Audits and Confidential Enquiries across the UK [MBRRACE] definitions (4). We also aimed to include studies of mothers at risk of preterm birth or those who had previously experienced preterm birth. Other types of perinatal bereavement, such as early pregnancy loss, *in utero* death, antepartum stillbirth, or infant death, were ineligible as perinatal bereavement experiences are known to be different (17), and the focus of this review was perinatal bereavement as a result of a preterm labour and birth. No studies were excluded on the basis of design or language. Reviews (be they critical, narrative, scoping, or systematic) were excluded, although the reference lists of relevant reviews were hand-searched for any missed references. For a full list of inclusion and exclusion criteria, please see the published protocol (15) or **Table 1**.

**Table 1:** Inclusion and exclusion criteria.

Inclusion Criteria	Exclusion Criteria
- Mothers of a premature infant (born up to 36^+6^ weeks) who died as a result of either neonatal death, early neonatal death, or late neonatal death (defined according to MBRRACE definitions) - Mothers who experienced an intrapartum stillbirth (a baby delivered at or after 24 completed weeks’ gestational age showing no signs of life and known to have been alive at the onset of care in labour) - Mothers of singletons or multiple birth - Studies which included a term sample, if a premature sample was also present and used as a comparison - Mothers at risk of preterm birth - Mothers who have previously experienced preterm birth (and still meet the first or second inclusion criteria)	- Mothers of infants who were not born prematurely or who die after the first 28 days of life - Other types of perinatal bereavement, such as early pregnancy loss (the loss of a pregnancy before 14^+0^ weeks’ gestation) and antepartum foetal death, infant death

### 2.4 Information sources

Searches included both peer-reviewed literature as well as grey literature and theses/dissertations. All databases (British Library EThOS; Cochrane Library; CINAHL; MEDLINE (Ovid); OPENGREY; ProQuest; PsycArticles; PsycINFO; PubMed; Scopus) were searched from inception to 5 April 2024, and included psychological, medical, and clinical databases (initially we also had intended to search on Embase, however Scopus includes all articles covered by Embase, rendering a separate search unnecessary) (15). The initial search strategy published on PROSPERO was expanded because the initial strategy was deemed too narrow after searches were conducted; the expanded strategy was added as a revision to the initial protocol (15) and can also be found in **Supplementary Table 1**.

### 2.5 Selection process

Searches were conducted by one author [SW] and exported to Rayaan (18), a commonly used web-tool to assist with the screening of articles against inclusion and exclusion criteria. All results were screened by title, abstract, and full text by two authors [SW, OP]. Any conflicts which could not be resolved between the screening authors were referred to a third author [SAS].

### 2.6 Data collection process and data items

Full data extraction for all reports was conducted via a pre-prepared Microsoft Excel spreadsheet, containing sub-headings to extract information. For the sub-headings, please see the published protocol (15).

### 2.7 Synthesis methods

Quantitative studies were to be synthesised using a convergent integrated approach (19), whereby data would be transformed into a compatible format to be analysed. The attribution of codes and/or themes to quantitative data is less likely to produce errors compared to assigning numerical values to qualitative data (i.e., ‘qualitizing’ the quantitative) (20). Studies were then to be synthesised and presented using narrative synthesis (21).

Qualitative studies were also to be synthesised and presented using narrative synthesis (21). An inductive multi-stage approach was to be used to collate narrative findings of each study, once exported to NVivo15. Results would be coded according to their meaning and content. Descriptive themes would be developed, and a hierarchical structure was to be built, by considering similarities and differences between codes, with the aim of developing analytical themes in a consultative and iterative way.

### 2.8 Reporting bias assessment

All eligible studies were to be independently assessed using the Critical Appraisal Skills Programme [CASP] Tool (22). CASP includes various checklists for a range of study designs, including quantitative, qualitative and case control studies. Reports and articles are rated on a ‘yes’, ‘no’, and ‘can’t tell’ system, with the ability to add further comments. No studies would be excluded based on a low rating. Bias assessment was to be used to aid data synthesis and interpretation and weighting of results.

## 3 Results

### 3.1 Screening

In total, 1,244 citations were extracted from the databases (see **Figure 1**). Suspected duplicates were identified automatically in Rayaan and verified by one author [SW]. Of the 576 articles reviewed at title and abstract stage, only 44 articles were reviewed as full text. Of these, seventeen were longitudinal studies (23–39), five were qualitative studies (40–44), twelve were cross-sectional or case control studies (45–56), five were literature reviews (57–61) and one was a study protocol (62). When contacting for further clarification, the author of the study protocol identified four further articles (63–66) that were published following the protocol which were reviewed at full-text stage, that used data from a cohort study.

No studies were eligible for inclusion in the review, rendering it an empty review. When there was a lack of clarity in the published article and so a decision on eligibility could not be made with certainty, one author [SW] contacted the authors of the original paper to provide further information. Initially, contact was made via e-mail if available, which was done throughout August and September 2024. Each author was contacted at least three times. When e-mail addresses were unavailable, other methods of contact were attempted, including via ResearchGate, and other members of the authorship team, or wider research teams, if their e-mails were also available. No study was excluded solely on the basis of the author not responding to our contact; in most cases it was that no information on type of stillbirth or gestational age was recorded, which they informed us via e-mail. One article was translated from German to English (58). The most common reasons for exclusion included: no information on whether the stillbirth was intrapartum or antepartum (n=7) (24, 28–30, 34, 46, 55), stillbirth or neonatal death were grouped with other types of loss and/or analysed as one variable (n=6) (27, 45, 47, 51, 53, 54,) stillbirths were not intrapartum only (n=6) (23, 36, 37, 40, 42, 49). For a full list of all studies and the reason for exclusion, see **Table 2**.

**Table 2:** List of studies and reason(s) for exclusion (N=44).

Author and year	Reason(s) for Exclusion
Accortt et al. (2022) (32)	No association between preterm birth and intrapartum stillbirth or neonatal death was analysed Gestational age was recorded but some infants of parents with adverse perinatal outcomes were born at term
Arach et al. (2020) (49)*	Stillbirth and neonatal death were grouped as one outcome variable, and stillbirths were both antepartum and intrapartum
Armstrong & Hutti (1997) (45)**	Previous miscarriage, stillbirth, and neonatal death were grouped as one variable Gestational age of previous loss was not recorded
Benfield et al. (1976) (48)	Mothers who had experienced intrapartum stillbirth or neonatal death were not included
Bohn (2023) (57)	Literature review
Brintow et al. (2023) (63)*	Gestational age was recorded, but not all were preterm, and the sample was not split by gestational age
Burkhammer et al. (2003) (40)	Previous stillbirth was not intrapartum
Cambonie et al. (2023) (31)	Although the mean gestational age for both groups were premature, some infants were born at term (upper median for the withhold/withdraw group was 38 gestational weeks) and the analysis was not split by gestational age
Christiansen et al. (2013) (50)	Gestational age is reported but not all of the sample were preterm
Côté-Arsenault & Dombeck, (2001) (47)*	Definition of perinatal loss included any type of loss Gestational age of previous loss was recorded, but not all of the previous losses were born preterm
Côté-Arsenault (2007) (28)*	Gestational age of previous loss was defined as ‘any’ so it was unclear if all of the sample were born preterm, or if the loss was through antepartum or intrapartum stillbirth
Couto et al. (2009) (51)	Women who had experienced loss were grouped with other adverse pregnancy outcomes (recurrent abortion, foetal death, preterm birth or early neonatal death) and analysed as one variable Gestational age of previous losses was not reported
Eklund et al. (2022) (65)*	Outcome variable was not psychological experiences
Engelhard et al. (2006) (25)**	In 95% of cases, the loss occurred before 12 weeks – the gestation of the remaining 5% was unclear
Garel et al. (2023) (44)	Some mothers did have experienced of previous losses, but the analysis was not split by these mothers alone
Gold et al. (2014) (30)*	Did not have information on whether stillbirth was antepartum or intrapartum Gestational age was unclear
Gravensteen et al. (2018) (36)**	Not clear if the stillbirth was antepartum or intrapartum
Hendy et al. (2024) (52)*	Although the author confirmed that all previous losses were born prematurely, the study considered both mothers and fathers and did not split their analysis by parent
Horsch et al. (2015) (37)*	All were antepartum stillbirths
Hunfeld et al. (1997) (38)	Not all of the sample were born preterm (range 24–38 weeks gestation) and in women who had experienced neonatal death, one infant died after 28 days of birth – the analysis was not separated by gestational age
Hvidtjørn et al. (2018) (62)*	Study protocol
Janssen et al. (1996) (27)**	Miscarriage and perinatal loss were grouped as one variable Gestational age of previous losses was recorded but there was no upper limit, so it is unclear if all the sample were preterm
Jørgensen et al. (2022) (66)*	Gestational age was recorded, but not all were preterm, and the sample was not split by gestational age
Kavanaugh & Robertson (1999) (42)	Losses in previous pregnancies were a mix of antepartum and intrapartum stillbirths, and although some of the analysis was focused on the most recent pregnancy, in which the participant went into preterm labour and the infant was stillborn, the focus of the study was not the psychological impact of that experience
Kelley & Trinidad (2012) (41)*	Was a secondary data analysis and gestational age data was not collected for the subgroup that was analysed
Kersting (2012) (58)	Literature review
Larsen (2022) (39)*	Investigated maternal depression as a risk factor for perinatal loss, rather than as an outcome variable Did not distinguish between antepartum and intrapartum stillbirth
Layne et al. (1990) (60)*	Literature review (does mention preliminary findings but did not have enough information for inclusion in the review)
Lewkowitz et al. (2019) (34)*	Information on whether the stillbirth was antepartum or intrapartum was not recorded
Mainali et al. (2023) (55)*	Did not record whether the stillbirth was antepartum or intrapartum Gestational age of stillbirth babies was not recorded
Mørk et al. (2023) (64)*	Gestational age was recorded, but not all were preterm, and the sample was not split by gestational age
Ng et al. (2020) (56)	Did not assess the association between preterm birth and intrapartum stillbirth or neonatal death and psychological experiences
Nilsson et al. (2001) (26)*	Analysed pregnancy outcomes in women with schizophrenia rather than psychological experiences after the loss
Ozdil (2023) (54)	Grouped women with previous miscarriage/stillbirth/neonatal death together Gestational age of previous death not reported
Prasad et al. (2022) (53)	Gestational age was reported, but adverse perinatal outcomes were grouped as women experiencing stillbirth, neonatal mortality, or neonate needing Neonatal Intensive Care Unit care
Redshaw et al. (2016) (23)**	Stillbirth was not intrapartum only Gestational age is reported but not all of the sample were preterm, and the data is not split by gestational age
Rosenbaum et al. (2011) (61)	Literature review (some case studies are presented, one of which features a preterm infant but they died after the first 28 days of life)
Shapiro et al. (2017) (29)*	Unclear if the stillbirths were antepartum or intrapartum
Shelkowitz et al. (2015) (46)**	Unclear if the stillbirths were antepartum or intrapartum Only 12 infants were born prematurely Mothers who had experienced stillbirth or neonatal loss (live birth of any gestational age or stillbirth over 20 weeks’ gestation) were grouped together
Sturrock & Louw (2013) (43)*	One participant in the sample carried to term, and the sample was not split by gestational age
Thomas et al. (2021) (33)*	Gestation of the previous stillbirth was not recorded
Treyvaud et al. (2016) (24)**	Unclear if the infant death was either by intrapartum stillbirth or neonatal death in the first 28 days of life
Van Dinter & Graves (2012) (59)	Literature review
Youngblut & Brooten (2018) (35)**	Gestational age data was not recorded Cause of death included conditions other than stillbirth

*Author was contacted and replied to our request for clarification or more information.

**Author was contacted, but did not respond to our request for clarification or more information.

*N.B.* In some cases, there is more than one reason a study does not meet the inclusion criteria of the review, however, exclusion decisions are listed according to the first criterion of exclusion, corresponding with that on the PRISMA 2020 diagram.

### 3.2 Reconciling the ‘empty’ systematic review

Despite no studies being eligible for inclusion in the review, they can still provide useful information. As it was not possible to answer the original research questions, in a deviation from the protocol, we applied less stringent criteria and extracted data from 19 of the above studies (23, 24, 27–30, 34, 36, 37, 40, 42, 45–47, 49, 51, 53–55), based on the most common reasons for exclusion: no information regarding stillbirth being intrapartum or antepartum (n=7), stillbirth or neonatal death were grouped with other types of loss and/or analysed as one variable (n=6), stillbirths were not intrapartum only (n=6); in order to provide information on psychological outcomes following perinatal bereavement. Where reported, studies were predominantly conducted in the USA (24, 28), and UK (23, 37), with the exact locations of other studies not being reported.

When combined, these studies indicated that the psychological impacts of perinatal loss could be pronounced up to 18 months after the loss, but inconsistent control for confounders and heterogeneity in timing of measure might limit the interpretation. The methodological quality of the studies was generally high, although more consistent reporting of gestational age in particular is needed for future studies. For full data extraction and critical appraisal of these studies, please see **Supplementary Tables 2**-**6**.

One study (24) investigated women with multiple birth whose children had died between two and seven years after birth. However, it was not included because there was no information on whether the stillbirth was intrapartum or antepartum. Nevertheless, the authors (24) demonstrated maternal mental health was similar in both mothers of multiples compared to singletons, but those who had experienced bereavement were 3.6 times more likely to have anxiety and depressive symptoms when their surviving child was seven years old. Similarly, another study (52) was excluded because both mothers and fathers were included in the analysis, and it was not split by parent. Nevertheless, it demonstrated parents who previously suffered a neonatal death and who had an infant in the Neonatal Intensive Care Unit [NICU] had significantly higher stress levels compared to those who had not suffered one (OR=3.21, 95%CI 0.96 to 10.73, p=.0470), although the sample size is small and the wide confidence intervals limit the interpretation of the findings.

Although excluded because all stillbirths were antepartum, one study (37) considering stillbirth also showed levels of PTSD were highest in the three months following a stillbirth. Furthermore, although excluded as the type of stillbirth was unclear, another study (34) highlighted within one year of stillbirth at >23 weeks’ gestation, women were nearly 2.5 times more likely to be re-admitted to hospital due to psychiatric morbidity (OR=2.47, 95%CI 2.20 to 2.77). Alongside this, the risks for postpartum psychiatric illness were highest within four months of suffering a stillbirth (aHR=3.26, 95%CI 2.63-4.04) (34). Similarly, despite being excluded in part because stillbirths were both antepartum and intrapartum, one study (49) found that over half (62.3%) of women who had experienced a perinatal death were classified as having probable depression based on an Edinburgh Postnatal Depression Scale [EPDS] cut-off of >14. Although excluded because there was no information on the type of stillbirth or gestational age, another study (30), found that in the adjusted analysis bereaved mothers had more than twice the odds for GAD (OR=2.39, CI(1.10-5.18), p=.28) and social phobia (OR=2.32, CI=1.52-3.54), p<.0005, but not panic disorder (p=.214) or OCD (p=.112). Further, despite the type of stillbirth being unclear, it has been found that following a previous stillbirth women in their current pregnancy were more likely to have anxiety compared with the previous live birth group (aOR=5.47, 95%CI= 2.90-10.32, p<.001) and the previously nulliparous group (aOR=4.97, 95%CI 2.68-9.24, p<.001) after adjusting for demographic factors (36). Additionally, they were significantly more depressed (aOR=1.91, 95%CI= 1.11-3.27, p=.019) than the previous live birth and the nulliparous group (aOR=1.91, 95%CI=1.11-3.36, p=.026) after adjusting for demographic factors (36). Finally, despite being excluded because the type of stillbirth was unclear, one study (29) found that after adjusting for all variables, prior stillbirth was not significantly associated with pregnancy anxiety in the first (β=0.30, 95%CI=0.10-0.70), p=.13), or second (β=0.24, 95%CI=0.08-0.57, p=.14) trimester, but was in the third trimester (β=0.40, 95%CI-0.05-0.74, p=.025).

## 4 Discussion

### 4.1 Main findings

The aim of this systematic review was to understand the psychological experiences of mothers who begin labour prematurely and whose babies subsequently died. Inclusion criteria aimed to identify women who had experienced intrapartum stillbirth or neonatal death with a preterm baby. No studies met criteria for inclusion in the review. Whilst frustrating to have a systematic review rendered empty, this demonstrates two needs from the field of research. Firstly, this empty systematic review demonstrates the need to accurately document data on the gestational age at which a baby was stillborn or died. Secondly, research must ensure perinatal bereavements are not grouped, but documented separately and accurately (17). Finally, this empty systematic review can be used as a call for future research conducted with women who go into labour prematurely to ensure psychometric measures of psychological health and experiential qualitative data are captured when including women who subsequently experience a perinatal bereavement.

### 4.2 Interpretation of findings

It is well established that women who experience stillbirth, regardless of gestational age, are more likely to experience poor mental health for a prolonged period after the death (67). However, the results of the review highlight that women who go into preterm labour with a baby who dies during active labour (i.e., intrapartum stillbirth) are an under researched group. Seven studies were excluded from the review because there was no information on whether the stillbirth was intrapartum or antepartum (24, 28–30, 34, 46, 55), and six studies were excluded because stillbirth or neonatal death were grouped with other types of loss and/or analysed as one variable (27, 45, 47, 51, 53, 54). This is surprising given preterm delivery has been identified as an independent risk factor for intrapartum foetal death (68). Grouping stillbirth and neonatal death with other types of loss, such as earlier pregnancy losses, is problematic in the context of preterm birth as women may experience these differently (69). Other types of *in utero* death, such as pregnancy losses of all kinds and antepartum stillbirths, present distinctly different lived experiences, as no labour and birth to a live infant is part of the pregnancy and birthing journey. Furthermore, stillbirth has been associated with feelings of ‘failure’ and guilt surrounding the self (70) whereas neonatal death has also been associated with increased anxiety surrounding the fragility of the infant, which is already well established in the context of preterm birth (71). Although in some cases this was the aim of the studies, it is important in future studies to further distinguish between stillbirth and other types of loss in the first instance, but also between antepartum and intrapartum stillbirth, particularly as the two can have differing incidence and causes (72), so psychological outcomes may vary. A recent study (73) examining stillbirth using gestational age in a sample of over 125 million births indicated approximately 74% of stillbirths were preterm, but also called for greater granularity in assessing risk, including by gestational age categories and labour types to provide greater understanding.

Four studies (25, 31, 38, 50) were excluded from the review because the analysis was not split by gestational age or the gestational age was unclear. Another four studies (33, 35, 41, 43) were excluded because gestational age data were not available. The disparity in reporting of gestational age, both in the case of stillbirth and neonatal death means that, despite the increased risk of perinatal loss associated with preterm birth, this group is underrepresented, and in some cases completely missed, in current research. It is already well known that the birth of a preterm baby can lead to an increased likelihood of developing maternal mental health difficulties, and can lead to feelings of detachment towards the infant, anxiety surrounding their health, and fear surrounding their survival (74). Coping with these emotions whilst dealing with the increased likelihood of the loss of an infant, or the ambiguity surrounding potential end-of-life decisions, alongside complex medical difficulties may lead to difficulties expressing these emotions (75). Studies have found that parents who feel more involved in their infant’s care whilst in the NICU may help families to navigate their grief (76). Although the majority of admissions to the NICU are for term infants (77), mothers of premature infants are a particularly important group to consider because their experiences may be further compounded by the unexpectedness, and potentially traumatic nature, of the birth (78). Future studies considering experiences of stillbirth or neonatal death should consider routine reporting of gestational age to enable greater understanding of the experiences of mothers who give birth preterm to a baby who then subsequently dies.

### 4.3 Strengths, limitations, and future directions

This comprehensive systematic review searched a range of clinical, psychological, and medical databases, with all screening decisions assessed independently by two reviewers, ensuring robustness. Despite potential criticisms that empty systematic reviews result from overly niche topics, we expanded the initial search strategy following patient and public involvement and engagement consultation, as well as preliminary searches, to broaden the focus.

An empty systematic review, whilst initially appearing unproductive, holds substantial significance in the scientific community by highlighting critical gaps in the existing literature (79, 80). Rather than merely indicating a lack of data, it highlights areas where evidence is lacking, guiding researchers to design studies that address these deficiencies and informing funding agencies about the importance of investing in under-researched topics. Empty reviews influence various stakeholders by encouraging researchers to conduct studies addressing the identified gaps, thus preventing duplication of effort and fostering a cumulative knowledge-base (80). For clinicians, awareness of the lack of evidence informs practice, highlighting the need for caution or alternative approaches when evidence is insufficient (79). Policymakers can identify areas where guidelines may need development or revision, ensuring that policies are based on comprehensive and current evidence.

Additionally, conducting research with vulnerable populations, such as women who have experienced preterm labour resulting in intrapartum stillbirth or neonatal death, presents ethical challenges (81, 82). Emotional distress, fear of re-living trauma, and protective gatekeeping can hinder participant recruitment. To overcome these barriers, researchers should emphasise beneficence by ensuring that the research is designed to provide potential benefits to participants or contribute valuable knowledge (81). In practice, this could be done by implementing sensitive consent processes which use compassionate communication strategies as respecting the emotional state of participants is crucial (82). Engaging with support groups and patient advocates, as demonstrated in the process of conducting this review, can build trust and facilitate recruitment by collaborating with organisations that support bereaved mothers. It has been recently identified that whilst mothers who have experienced a loss are generally very keen to take part in research, a key barrier is not understanding how they can take part or if their reasons for taking part will be appreciated (83). As such, similar to other studies, researchers could consider developing guides for taking part in research surrounding perinatal loss, to encourage participation and open up dialogue between researchers and participants (83).

To address the identified gaps, future research should consider alternative methods or broader inclusion criteria to capture relevant data. Expanding criteria to include various gestational ages or related experiences is also necessary to gather more comprehensive data. Emphasising the importance of reporting gestational age and distinguishing between types of perinatal loss will enhance the specificity of research findings. By addressing these limitations and implementing alternative approaches, future research can effectively fill the gaps identified by this empty review, ultimately contributing to better support and interventions for bereaved mothers.

## 5 Conclusion

This systematic review aimed to understand the psychological experience of mothers who gave birth prematurely to a baby who subsequently died. Whilst preterm birth only occurs in approximately 10% of cases, it is the leading cause of neonatal death in the UK. No studies were eligible for inclusion in the review. Reasons for exclusion included lack of clarity or distinction between antepartum and intrapartum stillbirth, or disparity in reporting of gestational age. Therefore, this review highlights the lack of research and understanding surrounding experiences of mothers who experience the loss of a premature baby and emphasises the need for greater research and reporting of gestational age in this context.

## 6 Definitions

*Gestational Age:* The number of weeks of age of the foetus or newborn infant, based on the time from the mother’s last menstrual period until the present date. *Perinatal Period:* The time between conception and up until the end of the first postpartum year. *Perinatal Bereavement:* Any form of pregnancy loss or perinatal death (including stillbirth and neonatal death). *Thanatophobia:* Extreme fear of death or the dying process. *Preterm Birth:* Birth at less than 37 weeks’ gestation. *Extremely Preterm:* Birth at less than 28 weeks’ gestation. *Very Preterm:* Birth at 28 to less than 32 weeks’ gestation. *Moderate to Late Preterm:* Birth at 32 to 37 weeks’ gestation. *Stillbirth:* A baby delivered at or after 24 completed weeks’ gestational age showing no signs of life, irrespective of when the death occurred. *Antepartum Stillbirth:* A baby delivered at or after 24 completed weeks’ gestational age showing no signs of life and known to have died before the onset of care in labour. *Intrapartum Stillbirth:* A baby delivered at or after 24 completed weeks’ gestational age showing no signs of life and known to have been alive at the onset of care in labour. *Neonatal Death:* A liveborn baby (born at 20 completed weeks’ gestational age or later, or with a birthweight of 400g or more where an accurate estimate of gestation is not available), who died before 28 completed days after birth. *Early Neonatal Death:* A liveborn baby (born at 20 completed weeks’ gestational age or later, or with a birthweight of 400g or more where an accurate estimate of gestation is not available) who died before 7 completed days after birth. *Late Neonatal Death:* A liveborn baby (born at 20 completed weeks’ gestational age or later, or with a birthweight of 400g or more where an accurate estimate of gestation is not available) who died after 7 completed days but before 28 completed days after birth.
