# Supplementary material for: Women’s psychological experiences of preterm labour and birth which results in an intrapartum stillbirth or a neonatal death: an empty systematic review
Source: Front Psychiatry. 2025 Jun 5;16:1544485. doi: 10.3389/fpsyt.2025.1544485 (PMC12176837; doi:10.3389/fpsyt.2025.1544485)
Supplement: Supplementary file 1 [file DataSheet1.pdf]

Supplementary Material: Tables 1-6

Supplementary Material (Table 1): **Example of a search strategy used.**

**Search Term**
AB ("preterm birth" OR "premature birth" OR "prematurity" OR "gestational age" OR "neonate" OR "NICU" OR "neonatal intensive care unit" OR "low birth weight" OR "special baby care unit" OR "SBCU" OR "local neonatal unit" OR "special care unit" OR "SCU")
**AND**
AND AB ("neonatal death" OR "neonatal loss" OR "perinatal bereavement" OR "perinatal loss" OR "intrapartum stillbirth" OR "intrauterine death" OR "dead on arrival" OR "neonatal mortality" OR "fetal mortality" OR "foetal mortality" OR "fetal death" or "foetal death")
**AND**
AND AB ("postnatal anxiet*" OR "postpartum anxiet*" OR "maternal anxiet*" OR "perinatal anxiet*" OR "generalised anxiet*" OR "generalized anxiet*" OR "postnatal depress*" OR "postpartum depress*" OR "maternal depress*" OR "perinatal depress*" OR "stress" OR "traumatic" OR "post-traumatic" OR "perinatal mental health" OR "maternal mental health")

Supplementary Material (Table 2): **Data extraction for quantitative studies.**

Author and Year	Study Title	Study Aim	Research Question	Study Setting	Population Demographics	Gestational Age	Age at which the baby died	Sample Strategy/Size	Psychometric Measure Used	Study Design	Method of Data Collection	Ethical Issues	Method of Analysis (incl. control for confounders)	Study Findings
Arach et al., 2020	Perinatal death triples the prevalence of postpartum depression among women in Northern Uganda: A community-based cross-sectional study	Not given	Hypotheses: Women with perinatal death in Lira District were more likely to screen positive for postpartum depression than women without perinatal death	Lira, Northern Uganda	1124 (62.8%) had experienced perinatal death, 1392 (77.8%) primary education level, 913 (51.0%) parity between 1-4, 1633 (91.3%) married	Gestational age of previous loss not given	Gestational age of previous loss not given	N= 1789<br><br>n=77 peripartum deaths, 37 were stillbirths and 40 were early neonatal deaths<br><br>Convenience sampling	Edinburgh Postnatal Depression Scale<br><br>≥14 as a cut off	Cross-sectional (case control)<br><br>The study was nested in a randomised community-based trial, but for this study only PPD symptoms were compared among women with/without perinatal death	Participants who enrolled in the RCT and who gave birth at ≥28 weeks' gestation, and were available 50 days postpartum were eligible<br><br>Administered via interview	Not given	Adjusted for maternal age, maternal education, parity, wealth status, marital status, and place of birth<br><br>Multivariable analysis using Poisson estimator with log link	From the 77 women who experienced perinatal death, 48 (62.3%) [95%CI:50.81%,72.61%]screened positive for “probable depression” (EPDS scores ≥14)compared to 329 (19.21%)[95%CI:17.42%,21.15%] out of 1712 women with live infants.

														Women who had experienced a perinatal death were three times as likely to have probable postpartum depression as those who had had a live infant at day 50 postpartum (adjusted prevalence ratio=3.45, [95%CI:2.67,4.48])

Armstrong & Hutti, 1998	Pregnancy after perinatal loss: the relationship between anxiety and prenatal attachment	To determine the difference between levels of pregnancy-specific anxiety and prenatal attachment in a group of pregnant women who previously had a late pregnancy loss and a group of primiparous women of similar gestational age<br><br>To determine the relationship (if any) between anxiety and prenatal attachment in both groups	Not given	Medical offices, childbirth classes, and perinatal support groups and newsletters - country not explicitly stated	Previous loss group: Mean age=31.5, mean years of education=15.69, 56.3% employed<br><br>First pregnancy group: Mean age=26, mean years of education=14.67, 80% employed	Gestational age of previous loss not given	Gestational age of previous loss not given	Convenience sampling<br><br>31 mothers - 16 had experienced miscarriage in the 2nd trimester, stillbirth, or early neonatal death during a previous pregnancy - 15 experienced their first pregnancy of a similar gestational age	Prenatal Attachment Inventory<br><br>Pregnancy Outcome Questionnaire	Comparative descriptive design (case control)	Questionnaires were mailed to participants	None given	Kruskal-Wallis test was used to evaluate differences between groups for anxiety and attachment<br><br>Spearman's correlations were performed to determine the relationship between the levels of anxiety and prenatal attachment for the two groups, and the relationship between anxiety and attachment	Participants who had experienced loss had significantly higher levels of anxiety compared to participants who had not (p=.0482)<br><br>No significant correlations

Côté-Arsenault, 2007	Threat appraisal, coping, and emotions across pregnancy subsequent to perinatal loss	To test Lazarus' theory of stress, coping, and emotions, and to understand the patterns of threat appraisal, coping, and emotional states of women across pregnancy after perinatal loss	Not given	Not clear	Mean(SD) age=30.2(5.04), primarily married or partnered, average 2 years college education, majority professionals or administrative staff, 88% white	Mean(SD) gestational age of one or more losses= 11.1(5.15)	Not given	N=82<br><br>Convenience sampling - women 20 years and older with a history of spontaneous pregnancy loss at any gestational age, without a technologically induced pregnancy, and prior to feeling fetal movement	Assignment of fetal personhood<br><br>Stress - two visual analogue scales 0-100 assessing stress in life excluding pregnancy, and stress from pregnancy<br><br>Threat appraisal of pregnancy using the MTI<br><br>Coping - The Ways of Coping Check List - Revised<br><br>Emotional states - Multiple Affect Adjective Checklist - Revised & Pregnancy Anxiety Scale	Longitudinal	Data was collected at three timepoints (10 weeks of gestation, 20-25 weeks of gestation, and 30-35 weeks of gestation)	Not given	Pearson's correlations were analysed between all T1 measures<br><br>One-way repeated measures ANOVA compared each main variable with all three points - post-hoc t-tests<br><br>Path analysis to determine the causal effects amongst threat appraisal, coping, and emoitonal states at T1 only<br><br>Hierarchical regression calculated for T1 data only - IV= emotional coping/problem focused coping, DVS= pregnancy anxiety, negative affect,	Pregnancy anxiety was significantly correlated with threat appraisal ($r=.728$, $p\leq.01$), emotion-focused coping ($r=.419$, $p\leq.01$), negative affect ($r=.522$, $p\leq.01$), positive affect ($r=-.359$, $p\leq.01$), and stress from pregnancy ($r=.582$, $p\leq.01$). Threat appraisal was significantly associated with the above variables and mean gestational age of loss ($r=.222$, $p\leq.05$), and fetal personhood ($r=.307$, $p\leq.01$)<br><br>A significant effect of time was found with pregnancy anxiety ($F(2, 68)=12.95$, $p<.001$) - follow-up t-test revealed pregnancy anxiety significantly decreased over time. Time was not significantly associated with threat appraisal ($F(2,68)=1.02$, $p>.05$), problem-focused coping ($F(2,68)=2.3$,

													positive affect	p>.05), emotion-focused coping (F(2,68)=0.18, p>.05), relative coping (F(2,68)-0.40, p>,05), negative affect (F(2,68)=0.40, p>.05), or positive affect (F(2,68)=0.08, p>.05)<br><br>In the path analysis, threat appraisal was significantly associated with problem focused coping (p<u><</u>.05), negative affect (p<.05), positive affect (p<.05), and pregnancy anxiety (p<.05). Regression results indicate that threat appraisal significantly predicts pregnancy anxiety R2=.530, adjusted R2=.524, F(1,80)=90.32, p<.001<br><br>Problem-focused coping was the dominant form of coping at all time points (p<.01) - only the unique contribution of emotion focused coping on pregnancy

														anxiety (R^2=.18, p<.001) and dysphoria (R^2=.10, p=.003) were significant

Côté-Arsenault & Dombeck, 2001	Maternal assignment of fetal personhood to previous pregnancy loss: relationship to anxiety in the current pregnancy	Examine the relationship between state and pregnancy anxiety and maternal assignment of personhood to the fetus lost in a previous pregnancy	1. How are state and pregnancy anxiety in a current pregnancy related to maternal assignment of fetal personhood to her lost fetus and to (1) the gestational age of the fetus at the time of loss, (2) the number of perinatal losses, (3) the time since the perinatal loss, and (4) the number of living children	Private obstetrical offices<br><br>Exact location not clear	50 (68%) did not percieve their pregnancy as high risk, 31 (51%) 1 years since last loss, 56 (82%) gestational age of loss 1 between 2-13 weeks, 16 (89%) gestational age of loss 2 between 2-13 weeks	Between 17-28 weeks' gestation	1 years since last loss, 56 (82%) gestational age of loss 1 between 2-13 weeks, 16 (89%) gestational age of loss 2 between 2-13 weeks	N=74 women with history of one or two perinatal losses<br><br>Convenience sampling	State Trait Anxiety Inventory (STAI)<br><br>Pregnancy Anxiety Scale (PAS)<br><br>Fetal personhood not measured using a validated measure	Descriptive, cross-sectional study	Self-administered questionnaires were placed on prenatal charts of eligible women in eight obsetrical offices	None given	Pearson's correlations were obtained for all of the main study variables to examine relationships between those variables	Assignment of fetal personhood was not related to state anxiety for the frst or second losses<br><br>The assignment of fetal personhood to the first losswas positively and signifcantly correlated with the amount of pregnancy anxiety a woman was experiencing in the current pregnancy (r= .30; p<.01) There was no signifcant relationship with personhood of a second loss (r=.10, p≥05)

Couto et al., 2009	Quality of life, depression and anxiety among pregnant women with previous adverse pregnancy outcomes	To compare the quality of life and the prevalence of symptoms of anxiety and depression amongst women with and without adverse pregnancy outcomes	Not given	A University clinic for high risk pregnancies, a tertiary hospital, two municipal healthcare clinics, Campinas, Brazil	Previous adverse pregnancy outcomes (n=120): Mean(SD) age=30.3(5.9), Mean(SD) number of previous pregnancies= 3.7(1.6), 88 married, 73 white, 32 education >10 years<br><br>Controls (n=120): Mean(SD) age=27.6(5.9), Mean(SD) number of previous pregnancies=1.4(0.6), 75 married, 70 white, 46 education >10 years	Gestational age of previous loss not given	Gestational age of previous loss not given	Convenience sampling<br><br>N=240 (120 cases who were receiving specialist antenatal care, 120 controls receiving routine care) between 18-24 weeks<br><br>Power calculation was conducted	Short Form-36<br><br>Hospital Anxiety and Depression Scale (HADS)<br><br>Health Insurance Experience<br><br>Medical Outcomes Study	Analytical cross-sectional (case control)	Cases (women who had $\geq$3 spontaneous successive fetal losses before 20 weeks, fetal death (death of a product of conception prior to complete expulsion or extraction from its mother irrespective of the duration of pregnancy), preterm birth (delivery before 37 weeks), or early neonatal death (death of a live-born baby within the first seven days of life)<br><br>At appointments, medical records examined to check if they had previous adverse	Exclusion criteria included multiple pregnancy, polyhydraminos, morbid obesity, or history of mental disease	SF-36 from the two groups compared using Wilcoxon signed ranks test<br><br>HADs evaluated using chi-square	Anxiety (p<.0001) and depression (p<.0001) were significantly higher in women with adverse pregnancy outcomes

											outcomes and invited to take part in an interview to collect the data			

Supplementary Material

Gold et al., 2014	Anxiety disorders and obsessive compulsive disorder 9 months after perinatal loss	To assess the prevalence of anxiety disorders and obsessive compulsive disorder amongst bereaved and live-birth mothers	Not given	Michigan, USA	Mean(SD) maternal age at delivery=29(6), 214 (35%) some college but less than Bachelor's degree educated, 483 (79%) Caucasian, 322 (53%) past medical history of any psychiatric disorder	Not given	Not given (authors accessed either the baby's fetal death or infant death certificate), but surveys were completed a median of 9 months after the loss	Collaboration with the Michigan Department of Community Health - using information from birth, fetal death, or infant death certificates, mothers were mailed with information about the study<br><br>609 completed and eligible women - of these 232 (46%) of mothers had a live birth, 377 (42%) had a perinatal death	Generalized Anxiety Disorder Scale - 7-item [GAD-7] - scores of 10 or higher indicative of moderate or severe anxiety<br><br>Mini Social Phobia Inventory [MINI-SPIN] - 6 or above as positive<br><br>Primary Care Evaluation of Mental Disorders [PRIME-MD] - Patient Health Questionnaire Panic Module<br><br>Obsessive Compulsive Inventory (revised version) - 21 or above was a positive screen	Longitudinal study of mothers with perinatal death - defined as either a stillbirth (above 20 weeks of gestational age and at least 400g) or an early infant death (first 28 days of life)	Using birth certifcates and fetal and infant death certificates, Michigan Department of Community Health sent mailings to 900 bereaved mothers and 500 control mothers<br><br>Participants were surveyed at 6, 14, and 24 months after delivery - this study reports results from the 6 month wave	None given	Mental health outcomes for bereaved vs. nonbereaved mothers were analysed using χ^2 and logistic regression after controlling for maternal age, race, insurance type, level of education and days between the birth/loss and compeltion of the survey<br><br>Additional covariates were added based on the literature (current depression, prior psychiatric disorder, social support, intimate partner violence, current pregnancy	70 bereaved mothers (19%) had a positive screen for moderate or severe GAD, 164 (44%) for social phobia, 47 (12%) for panic disorder, and 35 (9%) for OCD - these were lower for nonbereaved mothers<br><br>In the adjusted analysis, bereaved mothers had significantly more than twice the odds for GAD (OR=2.39, CI (1.10-5.18), p=.028) and social phobia (OR=2.32, CI (1.52-3.54), p<.0005). Panic disorder (OR=1.55, CI(0.78-3.10), p=.214) and OCD (OR=2.20, CI (0.83=5.83), p=.112) were non-significant<br><br>There was no significant association between current pregnancy and anxiety disorder or OCD or associations with race

													status and mental health (bereaved mothers only))<br><br>Racial differences in positive screens and treatment rates of different subgroups analysed using χ^2	

Supplementary Material

Gravensteen et al., 2018	Anxiety, depression and relationship satisfaction in the pregnancy following stillbirth after the birth of a live-born baby: a prospective study	To investigate the prevalence of anxiety and depression in the pregnancy following stillbirth and assess gestational age at stillbirth and inter-pregnancy interval as individual risk factors<br><br>To assess the course of anxiety, depression and satisfaction with partner relationship up to 3 years after the birth of a live-born baby following stillbirth	Not given	Norway	Previous stillbirth (n=174): Mean(SD) maternal age=31.18 (4.63), 168 (97.7%) married/cohabiting, 67 (39.0%) low education, 32 (18.4%) previous miscarriage, 115 (68%) gestational age at stillbirth ?30 weeks<br><br>Previous live birth (n=362): Mean(SD) maternal age=31.29 (4.14), 356 (98.3%) married/cohabiting, 104 (29.1%) low education, 55 (15.2%) previous miscarriage<br><br>Previously nulliparous (n=365): Mean(SD) maternal age=28.70 (4.45), 345 (95.0%) married/co	In the previous stillbirth group, 115 (68.0%) >30 weeks gestational age at stillbirth	In the previous stillbirth group, 115 (68.0%) >30 weeks gestational age at stillbirth	Convenience sampling<br><br>N=901 total	Hopkins Symptom Checklist<br><br>Five item version of the Relationship Satisfaction Scale	Prospective cohort study	Postal invitation sent after registering for a routine ultrasound examination at 17 weeks - part of a wider Norwegian Mother and Child Cohort Study and on records from the Medical Birth Registry of Norway	None given	The McNemar's test was used to analyse the differences in frequency of anxiety, depression and relationship satisfaction between different time points<br><br>Binary and multivariate logistic regression models were used to estimate odds ratios (OR) and adjusted odds ratios (aOR) for anxiety and/or depression in subsequent pregnancy among women with a previous stillbirth compared with the two reference groups. Covariates that were unevenly distributed between	The aOR for anxiety was 5.47 compared with the previous live birth group (95% CI= 2.90–10.32,p< 0.001, adjusted for age, education, pre-pregnancy smoking and stressful life events) and 4.97 compared with the previously nulliparous group (95% CI 2.68–9.24,p< 0.001, adjusted for age and education)<br><br>The aOR for depression was 1.91 compared with the previous live birth group (95% CI 1.11–3.27,p= 0.019, adjusted for age, pre-pregnancy smoking, BMI and stressful life events) and 1.91 compared with the previously nulliparous group (95% CI 1.11–3.36,p=0.026, adjusted for age, education and BMI)<br><br>The proportion of women with both anxiety and depression in the third trimester was 12.7%

					habiting, 99 (27.9%) low education, 52 (14.2%) previos miscarriage								the groups (p< 0.1), associated with the outcome variable in a bivariate model (p< 0.1), and not strongly correlated (correlation coefficient < 0.7), were included in the multivariate analyses<br><br>For the stillbirth group, separate binary regression models were used to test if gestational age at stillbirth or interpregnancy interval were significant predictors foranxiety or depression in the subsequent pregnancy	among womenwith a previous stillbirth compared with 3.6% in each reference group (p< 0.001 for both comparisons)<br><br>The prevalence of anxiety and depression decreased significantly from first assessment to 6 months postpartum among women with a previous stillbirth (p< 0.001for anxiety and p= 0.031 for depression) - by six and 18 months postpartum, respectively, the prevalence of depression and anxiety was not significantly different between groups<br><br>From six to 36 months postpartum, the prevalence of anxiety and depression increased significantly in the stillbirth group (p= 0.039 and 0.035 respectively) and the prevalence of

														anxiety, but not depression, increased significantly in the nulliparous group (p= 0.039)<br><br>At 36 months postpartum, the prevalence of anxiety and depression was higher among women with a previous stillbirth compared with women with a previous livebirth, but not compared with previously nulliparous women.The prevalence of anxiety and depression in the third trimester differed among women with a previous stillbirth completing all five questionnaires compared with drop-outs at any point after 30 gestational weeks (for anxiety 15.2 vs 32.4%, respectively,p= 0.007 and for depression12.1 vs 29.7% respectively,p= 0.004)

Horsch et al., 2015	Cognitive predictors and risk factors of PTSD following stillbirth: a short-term longitudinal study	Not given	1. How do PTSD symptoms change from 3 to 6 months post loss? 2. What are cognitive predictors of PTSD symptoms at 3 and 6 months? 3. Do cognitive factors (appraisals, dysfunction strategies) of PTSD at 3 months explain changes in PTSD symptoms at 6 months 4. What are the relationships between risk factors (percieved social support, trauma history, obstetric history) and PTSD at 3 and 6 months?	England, UK	Mean(SD) age in years= 31.92(4.98), Mean(SD) number of living children= 0.51 (0.81), 56 (86.2%) white UK ethnicity, 40 (61.5%) married, Mean(SD) gestational age at stillbirth= 34.09 (5.95), Mean(SD) number of previous pregnancies= 2.15 (1.41), Mean(SD) number of previous perinatal losses= 0.38(0.80), Mean(SD) history of perinatal loss before stillbirth= 16 (24.6)	Mean(SD) gestational age at stillbirth= 34.09 (5.95)	Mean(SD) gestational age at stillbirth= 34.09 (5.95)	Consecutive sampling - experienced a stillbirth at 24 weeks gestational age or later<br><br>N=65	Structured Clinical Interview DSM-IV-PTSD<br><br>Posttraumatic Diagnostic Scale<br><br>Posttraumatic Cognitions Inventory<br><br>Responses to Intrusions Questionnaire<br><br>Social Provisions Scale		Study interviews - mixture of clinicial and self-report	None given	Pearsons correlations and multiple regression models	Both number of clinician-reported and frequency of self-reported reexperiencing, avoidance, and arousal symptoms decreased significantly between 3 and 6 months<br><br>Higher scores onperceived social support did not uniquely predict a decrease inthe number of reexperiencing, avoidance, or arousal symptoms (SCID) at 6 months (all p=ns). Mother’s age uniquely predicted a decline in number of clinician-reported avoidance symptoms (β=−.25,t=−2.02,df ad=54,p=.048). Having more children (β=−.32,t=−2.41,dfad=42,p=.021) and more previous pregnancies (β=−.31,t=−2.59,dfad=55,p=.012) predicted less clinician-reported

														avoidance symptoms at 6 months. For self-reported frequency of PTSD symptoms, higher total scores in perceived social support (β=−.26,t=−2.24,dfad=46,p=.030) predicted a decline in reexperiencing symptoms, whereas higher income (β=−.29,t=−2.67,df ad=51,p=.010) predicted a significant decline in arousal symptoms

Janssen et al., 1996	Controlled prospective study on the mental health of women following pregnancy loss	Hypotheses: women who experienced a loss would show more depression, anxiety, and somatisation	Not given	Netherlands and the Dutch-speaking part of Belgium	Women who lost a baby (n=227): 100 (44%) secondary education, 221 (98%) married or cohabiting, 91 (40%) had one living child<br><br>Comparison women (n=213): 96 (45%) secondary education, 209 (98%) married or cohabiting, 108 (51%) had one living child	Exact gestational age of previous losses not given<br><br>Reproductive history:<br><br>Women who lost a baby: 132 (58%) no pregnancy loss, 83 (37%) early pregnancy loss <20 weeks, 12 (5%) perinatal loss $\geq$20 weeks<br><br>Comparison women: 172 (81%) no pregnancy loss, 35 (16%) early pregnancy loss <20 weeks, 6 (3%) perinatal loss $\geq$20 weeks	Exact gestational age of previous losses not given	N=440<br><br>Convenience sampling	SCL-90 (Dutch version)	Cohort study	Participants were mailed the surveys - they were followed up at 12 weeks, 1, 6, 12, and 18 months after delivery<br><br>At the 1 month assessment, women were asked if they had experienced loss (and they were included in the loss group)	None given	Used both a MANOVA and a MANCOVA of post delivery/loss scores with pretest scores (first assessment) as covariates - history of reproductive loss used as the first factor and loss group vs. comparison group as the second factor	Women shortly after pregnancy loss (approx. 2.5 months) had more mental health symptoms than women who gave birth to living babies (F=18.43, DF=4, 395, p<.0005), higher depression (F=54.05, DF=1, 399, p<.0005), anxiety (F=32.45, DF=1,399, p<.0005), somatisation (F=49.99, DF=1, 399, p<.0005), and OCD (F=11.03, DF=1, 399, p<.001).<br><br>At 6 months, women who had experienced a loss had more mental health symptoms than women who gave birth to living babies (F= 3.84, DF=4, 389, p.004) including higher symptoms of depression (F=7.56, DF=1, 392, p<.006), anxiety (F=6.14, DF=1, 392, p<.01), and somatization (F=13.10, DF=1, 392, p<.0005)<br><br>At 12 and 18

														months the differences between groups were no longer significant<br><br>When the effect of time since the loss was taken into account, those women whose period of gestation had been relatively longer showed more symptoms of depression (r partial=0.19, DF=224, p=0.004, one-tailed), anxiety (r partial=0.19, DF=224, p=0.005, one-tailed), somatisation (r partial=0.16, DF=224, p=0.01, one-tailed), and obsessive-compulsive behavior (r partial=0.18, DF= 224, p=0.007, one-tailed) shortly after pregnancy loss

Lewkowitz et al., 2019	Association between stillbirth >23 weeks gestation and acute psychiatric illness within 1 year of delivery	To determine whether women have a higher risk of experiencing clinician-diagnosed psychiatric morbidity in the year after stillbirth vs. livebirth	Not given	Florida, USA	Stillborn singleton >23 weeks gestation: 6354 (76.6%) aged 18-34, 3189 (39%) black, 3177 (38.9%) white, 2714 (38.9%) income quartile 1 (poorest)<br><br>Liveborn singleton: 970,842 (81.3%) aged 18-34, 602,944 (51.2%) white, 313,859 (31.5%) income quartile 1 (poorest)	N=8293 stillborn at >23 weeks gestation<br><br>N= 1,194,758 liveborn singleton	Not given	Utilised data from the Florida State Inpatient Database and Emergency Department Database of the Agency for Healthcare Research and Quality's Healthcare Cost and Utilisation Project from 2005-2015<br><br>N=8293 stillborn at >23 weeks gestation N= 1,194,758 liveborn singleton	Presentation to the Emergency Department or readmission to an inpatient hospital for treatment of an acute psychiatric illness - included suicide attempt, depression, anxiety, psychosis, posttraumatic stress disorder, acute stress reaction, adjustment disorder<br><br>Secondary outcome included alcohol or recreational drug use or dependence<br><br>Based on ICD-9-CM codes	Retrospective cohort study	Participants were identified from hospital and state department databases using ICD-9-CM diagnosis to identify participants, exposures, and outcomes	Multiple gestation excluded from the analysis Preexisting psychiatric illness during pregnancy excluded	Multivariable logistic regression were adjusted for age, race/ethnicity, payer, income quartile by ZIP code, mode of delivery, maternal medical comorbidities, and severe intrapartum maternal morbidity<br><br>Cox proportional hazard ratios examined the associated between stillbirth and the primary outcome over the 12 month follow up<br><br>Outcomes also changed to include only hospital admissions to analyse the impact of stillbirth on inpatient	In the adjusted model, the risk of readmission or Emergency Department encounter with coding for psychiatric illness was almost 2.5 times higher after stillbirth at >23 weeks gestation (OR=2.47, 95%CI 2.20-2.77)<br><br>Women who had experienced stillbirth had higher risk of depression (OR=2.75, 95% CI 2.31-3.26) and anxiety (OR=2.29, 95%CI (1.93-2.70))<br><br>Women with stillbirth had a higher risk of being coded for drug or alcohol use dependence both inpatient and in the Emergency Department (OR=2.41, 95% CI 1.99-2.90)<br><br>Cox proportional hazards was only not violated when follow-up was limited to the first four months (p=.1) and 4-12 months

													psychiatric care	(p-.1) post discharge - development of the primary outcome after birth was significant for the first 4 months (HR=3.26 (95%CI 2.6-4.04), and 4-12 months (HR=2.42, 95%CI 2.13-2.76)<br><br>When Emergency Department encounters were excluded, inpatient hospitalisation was three times higher when women had experienced stillbirth (OR=2.81, 95%CI 2.37-3.33) - risk of adjustment disorder (OR=4.46, 95%CI 2.86-6.94) and PTSD nearly 4 times higher (OR=5.36, 95%CI 2.73-10.51)

Mainali et al., 2023	Anxiety and depression in pregnant women who have experienced a previous perinatal loss: a case-cohort study from Scandinavica	To explore the association between previous perinatal loss and anxiety/depression symptoms in the subsequent pregnancy<br><br>To explore possible determinants of maternal mental health during the subsequent pregnancy, independent of previous perinatal loss	Not given	Three Scandinavican<br><br>Universities in Norway (Trondheim, Bergen) and Sweden (Uppsala)	Mean(SD) maternal age=28.48 (4.2), 1420 (97.4%) married/cohabiting, 755 (51.8%) high school educated, 614 (42.1%) part time work, 1080 (74.1%) smoking	Gestational age of previous loss not given	Perinatal loss grouped and included spontaneous abortion, stillbirth, or neonatal death	Used data from the Successive Small-for-Gestational Age Births Study (SGA)<br><br>N=1458; cases=401 with previous perinatal loss, non-cases=1057 who did not report perinatal loss	State Trait Anxiety Inventory (STAI)<br><br>Centre for Epidemiological Studies Depression scale (CES-D)	Case-cohort design	The wider study included interviews, questionnaires, self-study forms, and clinical examinations at 17, 25, 33, 37 weeks' gestation, and at birth<br><br>Participants responded to the measures at 25 weeks of the subsequent pregnancy	None given	Multiple linear regression used to assess the association between previous perinatal loss and maternal mental health in a subsequent pregnancy after controlling for confounders	After adjusting for age, civil status, education, occupation, economic situation, ability to raise NOK 5000 in one week, smoking status, alcohol consumption, pregnancy intention, and previous history of mental health, there was a positive association between previous perinatal loss and total depression score (β=0.90, 95%CI 0.06-1.74) and total anxiety score (β=1.22, 95%CI 0.49-1.95)

Supplementary Material

Ozdil, 2023	Postpartum depression among mother of infants hospitalised in the neonatal intensive care unit during the COVID-19 pandemic	To evaluate postpartum depression and related factors in mothers of infants hospitalised in NICU during two distinct COVID-19 pandemic periods and examine any addition effect of the pandemic on the mental health of postpartum women	Not given	Atatürk City Hospital, Turkey	Early group (COVID-19 restrictions in place; n=125): Median(IQR) maternal age=28(23.5-32), 80 (64%) multiparous, 50 (40.5%) maternal secondary high school education, 110 (88.5%) middle income status<br><br>Late group (fewer COVID-19 cases; n=125): Median(IQR) maternal age=28(24.5-33), 86 (69%) multiparous, 49 (39%) maternal primary school education, 104 (83.5%) middle income status	Gestational age of previous loss not given	Gestational age of previous loss not given	N=250 (n=150 in each group) women whose newborns were in the NICU from Nov 2021 to June 2022<br><br>Sample strategy not clear	Edinburgh Postnatal Depression Scale (Turkish version)	Cross-sectional	Scale was administered face-to-face to postpartum women between 14-30 days - the early group completed the survey between November 2021 - February 2022 during the winter peak of COVID-19; the late group completed this between March and June 2022 when there were fewer cases, deaths, and restrictions on visitation	Women who had recently given birth but declined to participate, had mental retardation, had a history of substance abuse, had twics, or had children with severe hypoxic-ischemic encephalopathy were excluded	Continuous variables evaluated using t-tests or Mann-Whitney U<br><br>Categorical variables evaluated using chi-square or Fisher's exact test<br><br>EPDS cut off $\geq$13<br><br>Pearson's correlations used to define relationships with EPDS scores<br><br>Odds ratios of multiple risk factors were evaluated by multiple logistic regression	EPDS scores $\geq$13 were significantly associated with previous history of abortion/stillbirth and/or neonatal death (OR=1.641, 95%CI 1.009-2.669, p=.04)

Prasad et al., 2023	Comparison of EPDS scores among women with good neonatal outcomes and adverse neonatal outcomes	To see if specific groups of women would benefit from selective screening	Not given	Tertiary care hospital, India	Women with good neonatal outcomes (n=80): Mean(SD) age=26.56 (6.12), 31 (38.75%) higher education, 44 (55%) primigravida, Mean(SD) gestational age)=37.6(1.23)<br><br>Women with adverse neonatal outcomes (n=80): Mean(SD) age=25.96 (5.98), 19 (23.75%) higher education, 38 (47.5%) primigravida, Mean(SD) gestational age= 35.3(2.54)	Women with good neonatal outcomes: Mean(SD) gestational age)=37.6(1.23)<br><br>Women with adverse neonatal outcomes:<br><br>Mean(SD) gestational age= 35.3(2.54	Gestational age of previous loss not given	Not clear - recruited in the hospital<br><br>N=160 (80 in each group)<br><br>Adverse neonatal outcomes defined as women who experienced stillbirth or neonatal mortality or neonate needing NICU care	Edinburgh Postnatal Depression Scale	Case control study	Demographic details gaathered via medical records and the EPDS was administered to women when they came for ther their post delivery follow-up or in the postnatal ward (2-3 weeks after delivery)	Already diagnosed with some psychiatric illness or delivered outside or those who needed ICU care during the perinatal period were excluded	Unpaired t-tests for continuous variables and chi-square for discrete variables	EPDS score of more than 9 was significantly higher in women with adverse neonatal outcomes (p=.0488)

Redshaw et al., 2016	Impact of holding the baby following stillbirth on maternal mental health and well-being: findings from a national survey	To compare mental health and well-being outcomes at 3 and 9 months after the stillbirth among women who held or did not hold their baby	1. How many women saw or held their baby after stillbirth? 2. Who held their baby after stillbirth? Did demographic, clinical or care characteristics differ between women who held and did not hold their baby? 3. Did mental health and well-being outcomes differ between those who held or did not hold their baby? Were differences significant after adjustment for demographic, clinical and care characteristics? 4. What was the unique impact of	England, UK	Held the baby (N=394): 202 (81.1%) aged 30-39, 351 (85.6%) white, 357 (84.4%) married or with partner<br><br>Did not hold the baby (N=74): 47 (18.9%) aged 30-39, 16 (21.8%) black, 66 (15.6%) married or with partner	Held the baby: 173 (88.7%) 37+ weeks<br><br>Did not hold the baby: 22 (11.3%) 37+ weeks	Held the baby: 173 (88.7%) 37+ weeks<br><br>Did not hold the baby: 22 (11.3%) 37+ weeks	All women who had a registered stillbirth in England between January-March 2012 or June-August 2012 were identified by the ONS and sent a study pack between 6-9 months after the stillbirth	Psychological wellbeing reported through a symptom checklist based on national surveys - asked if they had experienced any of the symptoms following 3 months after birth and in the last few days	Secondary analysis of a postal population survey	Participants filled in the survey and some data was given by the ONS	None given	Multiple logistic regression used to adjust for differences between the two groups<br><br>Bivariate analyses using χ^2 described outcomes individually for those who held and saw the baby, only saw the baby, and neither held or saw the baby<br><br>Logistic regressions, adjusting for confounders, compared holding and seeing - separate subgroup analyses conducted on current pregnancy status, time between antepartum death and the birth, gestation	After adjusting for multiple pregnancy, ethnicity, and fertility treatment: 3 months after birth there were no statistically significant associations with mental and physical health 9 months after birth, only anxiety (OR=2.12, 95%CI 1.11-4.04, p<.05) and relationship difficulties with family (OR=5.33, 95%CI 1.26-22.53, p<.05) were statistically significant<br><br>After adjusting for multiple pregnancy, ethnicity, and fertility treatment: Holding the baby was significantly associated with anxiety 3 months after birth (OR=3.80, 95% CI 1.55-9.35, p<.01) and 9 months after birth (OR=4.29, 95% CI 1.49-12.39, p<.01) and relationship difficulties with family 3 months after birth

			holding the baby on mental health and well-being outcomes, in contrast to that attributable to only seeing the baby?<br>5. Was the impact of contact with the stillborn baby different according to current pregnancy status, or the condition of the baby (captured by time from antepartum death to birth, stillbirth gestation, presence of congenital abnormality)?										at stillbirth, congenital abnormality	(OR=3.52, 95% CI 1.05-11.75, p<.05)<br><br>Seeing the baby was significantly associated with anxiety 3 months after birth (OR=0.19, 95% CI 0.06 to 0.60, p<.01) and 9 months after birth (OR=0.27, 95% CI 0.07 - 0.98)

Shapiro et al., 2017	Previous pregnancy outcomes and subsequent pregnancy anxiety in a Quebec prospective cohort	To measure the associations between five past pregnancy outcomes (live preterm birth, live term birth, miscarriage at <20 weeks, stillbirth at ≥20 weeks, and elective abortion)	Not given	One clinical centre, Quebec, Canada	917 (39%) aged 30-34,<br>860 (36%) had 0 previous pregnancies, 1017 (45%) annual household income 50,000-99,999,<br>911 (39%) university degree,<br>1636 (69%) white,<br>2232 (95%) married or living with a partner	Not given	Gestational age of previous loss not given	Sample strategy unclear	Anxiety measured using the Pregnancy Related Anxiety Scale (Dunkel-Schetter)<br><br>Measure not named used to measure disorders like phobias, GAD, obsessions, compulsions and PTSD<br><br>10- and 4-item version of the Center for Epidemiological Studies Depression Scale (CESD) to measure depressive symptoms	Longitudinal cohort study	Data on maternal demographic characteristics and pregnancy history for each known previous pregnancy (including those <20 weeks) were collected retrospectively via interviewer-administered questionnaires at study entry<br><br>Pregnancy anxiety for the (subsequent) index pregnancy and depressive symptoms were measured by self-administered questionnaire at each of the three prenatal visits (8–14 weeks, 20–24 weeks, 32–35 weeks)	Exclusion criteria included current intravenous drug use, severe illness or life threatening conditions, and multiple gestation pregnancies	Anxiety and depression measures and response rates across different strata of the sample using univariate ANOVA<br><br>For multigravid women only, Pearson's correlations between previous pregnancy outcome and subsequent depression and anxiety measures at each of the three trimesters - those that were significant were tested using linear regressions Adjusted models were included all five previous pregnancy outcomes	The number of previous live term births was significantly associated with lower pregnancy anxiety in all three trimesters (first, r=-.12, p<.01; second, r=-0.10, p<.01; third, r=-0.09, p<.01). Prior stillbirth (first, r=0.08, p<.01; second, r=0.09, p<.01; third. r=0.09, p<.001) and elective abortion (first, r=0.11, p<.01; second, r=0.11, p<.01, third, r=0.08, p<.05) were associated with higher pregnancy anxiety in all three trimesters. Prior miscarriage (r=0.09, p<.01) and prior PTB (r=0.06, p<.05) were significantly associated with pregnancy anxiety in the first trimester<br><br>After adjusting for all variables, prior stillbirth (β=0.30, 95%CI=-0.10-0.70), p=0.13) was not significantly associated with pregnancy

											The anxiety disorders screening instrument was administered only once at the second-trimester assessment		Sensitivity analysis predicting pregnancy anxiety whilst controlling for depression and anxiety screening score	anxiety in the first trimester, or the second trimester (β=0.24, 95%CI=-0.08-0.57), p=0.14), but was in the third trimester (β=0.40, 95%CI=0.05-0.74, p=0.025)

Supplementary Material

Shelkowitz et al., 2015 (quantitative only)	Counseling for personal care options at neonatal end of life: a quantitative and qualitative parent survey	To explore the personal care options offered to parents as well as parental perceptions of the counselling they received regarding these options at their infant's end of life	Not given	Not clear	Neonate characteristics (n=28): Mean(SD) gestational age=28.6(6.8), 62.5% male, 24 (86%) singleton, 12 (43%) diagnosed with prematurity	Mean(SD) gestational age=28.6(6.8)	Not given	n=28 neonates Identified through medical records and contacted for recruitment via a letter	Open ended text boxes	Cross-sectional survey	Participants were sent a contact letter to complete the survey either online, paper, or telephone - other demographic and clinical data was obtained via chart review	Not given	Continuous variables were analysed by t-test or Wilcoxon<br><br>Categorical data were analysed using chi-square	36% of parents received counselling during the same visit or hospitalisation in which they learned about their infant's diagnosis or prognosis - 23% received this during the initial visit and at later visits - 27% of parents stated that they received no counselling

Treyvaud et al., 2016	The influence of multiple birth and bereavement on maternal and family outcomes 2 and 7 years after very preterm birth	To examine the influence of multiple birth (twins or triplets) on mental health, parenting stress, and family functioning at two and seven years corrected age<br><br>To investigate whether bereavement within the context of very preterm birth influenced maternal mental health, parenting stress, and family functioning	Not given	Royal Women's Hospital, Melbourne, Australia	Singletons (n=129): 85 (66%) primipara, 64% secondary education at two, 52% secondary education at seven, 34% professional occupation at two, 34% unskilled occupation at two, 85% intact family structure at two, 74% intact family structure at seven<br><br>Multiples (n=33): 34 (75%) primipara, 45% tertiary education at two, 57% tertiary education at seven, 58% professional occupation at two, 57% professional occupation	Infants born at <30 weeks' gestation or with a birthweight <1250g	Not given	Part of a broader longitudinal study<br><br>N=162 mothers	General Health Questionnaire (GHQ; two years) - score of 24 or more indicates clinically significant symptoms of mental health problems<br><br>Hospital Anxiety and Depression Scale (HADS; seven years) - scores in the 11-21 range classified as clinically significant<br><br>Parenting Stress Index (PSI)	Longitudinal observational study	Families completed questionnaires at two and seven years corrected age	None given	To examine whether multiple birth and bereavement were associated with maternal mental health and family functioning, separate linear and logistic regression models were fitted to each continuous and categorical outcome (multiple vs singleton; no bereavement vs bereavement)<br><br>To examine whether multiple birth and bereavement were associated with greater parenting stress, linear regression models were fitted at the child	At two years, there was no significant difference between mental health, parenting stress, or family functioning<br><br>At seven years, bereaved mothers repoted more anxiety symptoms after controlling for social risk β(95% CI)=2.60, 0.25-4.95, p=.03. They were more likely to report elevated symptoms of anxiety (OR(95% CI)=4.12, 1.17-14.48, p=.03) and depression (OR(95% CI)=4.67, 1.29-16.93) after controlling for concurrent social risk

					at seven, 97% intact family structure at two, 80% intact family structure at seven								level using Generalised Estimating Equations(GEEs)	

Supplementary Material (Table 3): **Data extraction for qualitative studies.**

Author and Year	Study Title	Study Aim	Research Question	Study Setting	Population Demographics	Gestational Age	Age at which the baby died	Sample Strategy /Size	Psychometric Measure Used (if applicable)	Study Design	Method of Data Collection	Ethical Issues	Method of Analysis	Summary of Experiences	Theoretical Framework	Theme(s) Identified	Methodological Comments
Burkhammer et al., 2003	Grief, anxiety, stillbirth, and perinatal problems: healing with kangaroo care	Not clear	Not given	Not explicitly stated	Maternal age= 23	27 weeks	28 weeks (antepartum stillbirth)	Case study (n=1)	N/A	Case study	Method not explicitly stated but mother was visited by researchers	Not given	Not given	"Kim" was a 23-year-old, single mother who experienced pregnancy-specific anxiety during her first pregnancy that ended with a stillbirth at 28 weeks.	Not given	None (was a case study with results presented narratively)	None

														Four months later, she became pregnant again and experienced feelings of anxiety, attachment, and hypervigilance mixed with grief. Her newborn son was placed skin to skin on her chest for the first time and Kim was overcome with memories of holding her stillborn son, which caused the research team to intervene. As she continued skin-to-skin breastfeeding, the stories			

														of her stillborn son continued, and she reported an improvement in breastfeeding ease and a stronger bond with her baby.			

Supplementary Material

Kavanaugh & Robertson, 1999	Recurrent perinatal loss: a case study	To examine the impact of recurrent perinatal loss on a low income African-American parent	Not given	Explicit location not given	Maternal age=30, experienced three perinatal losses (18 weeks, 26 weeks where preterm labour was experienced, 12 weeks gestation) - fifth pregnancy was fourth loss (25 weeks gestation with preterm labour)	Experienced three perinatal losses (18 weeks, 26 weeks where preterm labour was experienced, 12 weeks gestation) - fifth pregnancy was fourth loss (25 weeks gestation with preterm labour)	Experienced three perinatal losses (18 weeks, 26 weeks where preterm labour was experienced, 12 weeks gestation) - fifth pregnancy was fourth loss (25 weeks gestation with preterm labour)	n=1 (case study)<br><br>Part of a larger phenomenological study	N/A	Case study	Two open ended interviews were conducted in the mother's home between seven and nine weeks after the most recent loss	Interviews were limited to two hours and collected over at least two interview sessions	Framework focused on Lazarus and Folkman's theory of stress and coping - data was coded according to three categories from the framework (antecedents, mediating processes, immediate effects)	Results demonstrated that the prior perinatal losses were not critical components of the way the mother responded to her most recent loss. Perception of the care she received from healthcare providers and how this related to her experiences with her one living child who was born at the same gestational age was an important determinant in the response to the loss	Lazarus and Folkman's theory of stress and coping	Causal antecedents, mediating processes, immediate effects	None

Shelkowitz et al., 2015 (qualitative only)	Counseling for personal care options at neonatal end of life: a quantitative and qualitative parent survey	To explore the personal care options offered to parents as well as parental perceptions of the counselling they received regarding these options at their infant's end of life	Not given	Not clear	Neonate characteristics (n=28): Mean(SD) gestational age=28.6(6.8), 62.5% male, 24 (86%) singleton, 12 (43%) diagnosed with prematurity	Mean(SD) gestational age=28.6(6.8)	Not given	n=28 neonates Identified through medical records and contacted for recruitment via a letter	Open ended text boxes	Cross-sectional survey	Participants were sent a contact letter to complete the survey - other demographic and clinical data was obtained via chart review	Not given	Content analysis	Participants preferred lots of options when their infant was coming to the end of their life	Not given	Guidance by healthcare providers: Importance of receiving information to assist with decision making by being presented with choices and options<br><br>Making memories: Parents appreciated memories to take away with them as that was all they had - these included memories boxes, handprints, footprints, photos<br><br>Feeling cared for and respected:	None

																Importance of feeling cared for during a difficult time<br><br>Regrets/ wishes: Described feelings of regret over what they wished would have happened e.g., spending more time with the baby	

Supplementary Material (Table 4): **Critical appraisal for case control studies.**

	**Section A: Are the results of the study valid?**							**Section B: What are the results?**			**Section C: Will the results help locally?**	
	1. Did the study address a clearly focussed issue? **HINT: An issue can be 'focused' in terms of the population studied, whether the study tried to detect a beneficial or harmful effect, the risk factors studied**	2. Did the authors use an appropriate method to answer their question? **HINT: Consider is a case control study an appropriate way of answering the question under the circumstances, did it address the study question**	3. Were the cases recruited in an acceptable way? **HINT: We are looking for selection bias which might compromise validity of the findings, are the cases defined precisely, were the cases representative of a defined population (geographically and/or temporally), was there an established reliable system for selecting all the cases, are they incident or prevalent, is there something special about the cases, is the time frame of the study relevant to disease/exposure, was there a sufficient number of cases selected, was**	4. Were the controls selected in an acceptable way? **HINT: We are looking for selection bias which might compromise the generalisability of the findings, were the controls representative of the defined population (geographically and/or temporally), was there something special about the controls, was the non-response high, could non-respondents be different in any way, are they matched, population based or randomly selected, was there a sufficient number of**	5. Was the exposure accurately measured to minimise bias? **HINT: We are looking for measurement, recall or classification bias, was the exposure clearly defined and accurately measured, did the authors use subjective or objective measurements, do the measures truly reflect what they are supposed to measure (have they been validated), were the measurement methods similar in the cases and controls, did the study incorporate blinding where feasible, is the temporal relation**	6. (a) Aside from the experimental intervention, were the groups treated equally? **HINT: List the ones you think might be important, that the author may have missed, genetic, environmental, socio-economic**	6. (b) Have the authors take account of the potential confounding factors in the design and/or in their analysis? **HINT: Look for restriction in design, and techniques e.g., modelling, stratified-, regression-, or sensitivity analysis to correct, control or adjust for confounding factors**	7. How large was the treatment effect? **HINT: Consider what are the bottom line results, is the analysis appropriate to the design, how strong is the association between exposure and outcome (look at the odds ratio), are the results adjusted for confounding, and might confounding still explain the association, has adjustment made a big difference to the OR**	8. How precise was the estimate of the treatment effect? **HINT: Consider size of the p-value, size of the confidence intervals, have the authors considered all important variables, how was the effect of subjects refusing to participate evaluated**	9. Do you believe the results? **HINT: Consider big effect is hard to ignore! Can it be due to chance, bias, or confounding, are the design and methods of this study sufficiently flawed to make the results unreliable, consider Bradford-Hills criteria (e.g., time sequence, dose-response gradient, strength, biological plausibility)**	10. Can the results be applied to the local population? **HINT: Consider whether the subjects covered in the study could be significantly different from your population to cause concern, your local setting is likely to differ much from that of the study, can you quantify the local benefits and harms**	11. Do the results of this study fit with other available evidence? **HINT: Consider all the available evidence from RCT's, Systematic Reviews, Cohort Studies, and Case Control Studies as well, for consistency**

			**there a power calculation**	**controls selected**	**correct (does the exposure of interest precede the outcome)**							
Arach et al., 2020	Y	Y	Y<br><br>Power calculation was conducted	Y	Y	Y	Y	Analysis was appropriate, controlled for confounders	Confidence intervals were small	Yes	Y	Y
Armstrong & Hutti, 1998	Y	Y	Y<br><br>Power calculation was not conducted	Y	Y	Y	N<br><br>No consideration of confounders	Results not adjusted for confounding - just assessed whether there was a significant difference between the groups	Can't tell (only p-values reported)	Partially - confounders were not considered	Y	Y
Couto et al., 2009	Y	Y	Y<br><br>Power calculation conducted	Y	Y	Y	N	Results not adjusted for confounding – just assessed whether there was a significant difference between the groups	Can't tell (only p-values reported)	Partially – recruitment and design were robust but confounders were not considered	Y	Y
Janssen et al., 1996	Y	Y	Y<br><br>Power calculation was not conducted	Y	Y	Y	Y<br><br>Only considered reproductive history	Results only considered reproductive history - just assessed whether there was a significant difference between the groups	Confidence intervals not reported for these analyses	Yes	Y	Y

Mainali et al., 2023	Y	Y	Y<br><br>No power calculation was conducted	Y<br><br>Completed the same measures at the same gestational week in the same settings – only the experience of the loss was different	Y	Y<br><br>Questionnaires completed in the same gestational week	Y<br><br>Yes, but how variables were decided to be included is not clear	Analysis was appropriate to the design, but adjusting for the confounders did not make a big difference to the regression coefficients - confounding may go some way to explaining the results	Important variables appear to be considered, although ethnicity was not reported or controlled for<br>Size of confidence intervals are moderate	Yes - authors controlled for a wide range of confounders, cases and controls were selected appropriately	Y<br><br>Yes, but most participants were married/cohabiting and at least high school educated, with a good/medium economic situation	Y
Prasad et al., 2023	Y	Y	Y<br><br>Power calculation conducted	Y	Y	Y	N	Results not adjusted for confounding - just assessed whether there was a significant difference between the groups	Can't tell (only p-values reported)	Partially - confounders were not considered and only p-values were reported not the full statistics	Y	Y
N/B. **Y=Yes, N=No, CT=Can't Tell. Comments are included in the same box where applicable**												

Supplementary Material (Table 5): **Critical appraisal for cohort studies.**

	Section A: Are the results of the study valid?								Section B: What are the results?			Section C: Will the results help locally?		
	1. Did the study address a clearly focussed issue? **HINT: A question can be 'focused' in terms of the population studied, the risk factors studied, is it clear whether the study tried to detect a beneficial or harmful effect, the outcomes considered**	2. Was the cohort recruited in an acceptable way? **HINT: Look for selection bias which might compromise the generalisability of the findings, was the cohort representative of a defined population, was there something special about the cohort, was everybody included who should have been**	3. Was the exposure accurately measured to minimise bias? **HINT: Look for measurement or classification bias, did they use subjective or objective measurements, do the measurements truly reflect what you want them to (have they been validated), were all the subjects classified into exposure groups using the same procedure**	4. Was the outcome accurately measured to minimise bias? **HINT: Look for measurement or classification bias, did they use subjective or objective measurements, do the measurements truly reflect what you want them to (have they been validated), has a reliable system been established for detecting all the cases (for measuring disease occurrence), were the measurement methods similar in the**	5. (a) Have the authors identified all important confounding factors? **HINT: list the ones you think might be important, and ones the author missed**	5. (b) Have they taken account of the confounding factors in the design and/or analysis? **HINT: Look for restriction in design, and techniques e.g., modelling, stratified-, regression-, or sensitivity analysis to correct, control, or adjust for confounding factors**	6. (a) Was the follow up of subjects complete enough? **HINT: Consider the good or bad effects should have to be long enough to reveal themselves, the persons that are lost to follow-up may have different outcomes than those available for assessment, in an open or dynamic cohort was there anything special about the outcome of the people leaving, or the exposure of the people entering the cohort**	6. (b) Was the follow up of subjects long enough?	7. What are the results of this study? **HINT: Consider what are the bottom line results, have they reported the rate or the proportion between the exposed/unexposed, the ratio/rate difference, how strong is the association between exposure and outcome (RR), what is the absolute risk reduction (ARR)**	8. How precise are the results? **HINT: Look for the range of the confidence intervals, if given**	9. Do you believe the results? **HINT: Consider big effect is hard to ignore, can it be due to bias, chance, or confounding, are the design and methods of this study sufficiently flawed to make the results unreliable, Bradford Hills criteria (e.g., time sequence, dose-response gradient, biological plausibility, consistency)**	10. Can the results be applied to the local population? **HINT: Consider whether a cohort study was the appropriate methods to answer this question, the subjects covered in this study could be sufficiently different from your population to cause concern, your local setting is likely to differ much from that of the study, you can quantify the local benefits and harms**	11. Do the results of this study fit with other available evidence?	12. What are the implications of this study for practice? **HINT: Consider one observational study rarely provides sufficiently robust evidence to recommend changes to clinical practice or within health policy decision making, for certain questions, observational studies provide the only evidence, recommendations from observational studies are always stronger when supported by other evidence**

				**different groups, were the subjects and/or the outcome assessor blinded to the exposure (does this matter)**										
Côté-Arsenault, 2007	Y	Y	Y	Y	N<br><br>None identified	N<br><br>None were controlled for in the hierarchical regression analyses	Y<br><br>Those lost to follow up were not reported	Y	Pregnancy subsequent to loss is perceived as a threat, and this strongly predicted pregnancy anxiety. Pregnancy anxiety decreased over time, but threat appraisal and coping remained stable	CIs not necessary to report for these analyses	Can't tell - small sample size, no control for confounders	Y	Y	Y<br><br>Anxiety should be addressed at each prenatal visit, but most notably in the first trimester when it is likely to be highest
Côté-Arsenault & Dombeck, 2001	Y	Y	Y	Y	N<br><br>No confounders identified or controlled for	N<br><br>No confounders identified or controlled for			Assignment of personhood was significantly related to pregnancy anxiety and to the gestational age of the first loss but not to state anxiety	CIs not applicable to this analysis	Partially - small sample size but no control for confounders in the analysis so would need to be repeated	Y	Y	Y

Gold et al., 2014	Y	Y<br><br>Community health survey	Y	Y<br><br>Used validated measures of anxiety, social phobia and OCD	Y	Y<br><br>Wide range of confounders adjusted for	Y	Y	After adjusting for covariates, bereaved mothers had higher odds of moderate-severe GAD and social phobia, but not panic disorder or OCD	Moderate range of confidence intervals	Yes - wide range of confounders controlled for, large sample size, inclusion of a control group	Y	Y	Y<br><br>Monitor women during subsequent pregnancies and consider targeted interventions

Gravensteen et al., 2018	Y	Y	Y	Y	Y	Y<br><br>They differed for each analysis	Y	Y	Women pregnant after stillbirth had a higher prevalence of anxiety (22.5%) and depression (19.7%) compared with women with a previous live birth and previously nulliparous women. Gestational age at stillbirth (> 30 weeks) and inter-pregnancy interval < 12 months were not associated with depression and/or anxiety. Anxiety and depression decreased six to 18 months after the birth of a live-born baby, but increased again 36 months postpartum.	Moderate-to high range in CIs	Yes - large sample size, robust analyses controlling for confounders	Y	Y	Y

									Relationship satisfaction did not differ between groups.					

Horsch et al., 2015	Y	Y	Y	Y	N	N	Y	Y	PTSD symptoms decreased between 3 and 6 months. Positive relationship between rumination and concurrent frequency of PTSD symptoms. Negative Self-View and Negative World-View related positively and Self-Blame related negatively to concurrent number of PTSD symptoms. Suppression and Distraction predicted a decrease and Numbing predicted an increase in time-lagged number of PTSD symptoms. Risk factors for PTSD symptoms were	CIs not reported for these analyses	Partially – small sample size, no control for confounders, but robust analysis	Y	Y	Y

									younger age, lower income, fewer previous pregnancies, and poorer perceived social support					
Lewkowitz et al., 2019	Y	Y<br><br>Cohort was limited to Florida, but the cohort was clearly defined and reasons for exclusion were given	Y<br><br>Coded using ICD criteria	Y<br><br>Unclear if the outcome assessor was blinded to the exposure	Y<br><br>Psychiatric disorders during pregnancy were excluded from the study	Y<br><br>Potential confounds controlled for and sensitivity analysis conducted	Y	Y<br><br>12 months	Women who have experienced stillbirth are more than twice as likely to have psychiatric morbidity compared to women who give birth to a live infant. They also are at increased risk of inpatient psychiatric care or Emergency Department admission - results suggest the highest risk interval for psychiatric illness is within the first 4 months after stillbirth	Range of confidence intervals is generally quite small, but were larger for the hazard ratios analysis	Yes - the analysis controlled for a wide range of confounding variables, data were from a hospital and state database, also conducted a sensitivity analysis	Y<br><br>Yes but was conducted in a high income country, participants were predominantly of low SES	Y	Y<br><br>Recommends to screen all women for depression and anxiety after delivery, but to provide individualised care within the first 3 months

Ozdil et al., 2023	Y	Y	Y	Y	N<br><br>No confounders identified or controlled for	N<br><br>No confounders identified or controlled for			EPDS scores significantly higher in women with a history of perinatal loss	CIs are very large	Partially - moderate sample size but no control for confounders in the analysis so would need to be repeated	Y	Y	Y<br><br>Suggests screening for depression whilst in NICU
Redshaw et al., 2016	Y	Y<br><br>Efforts were made to diversify the sample (i.e., survey offered in 18 different languages)	Y	N<br><br>Although questions about psychological wellbeing were based on other national surveys, the questions were not clear, and it was unclear if the questionnaires had been validated or were appropriate for the population	N<br><br>Only three confounders were controlled for, and it was unclear how these were identified to be included in the analysis	Y	Y	Y	Women who held their stillborn baby had higher rates of mental health and relationship difficulties	Confidence intervals are large - the authors suggest this is because a very low number of women saw (and did not hold) their baby	Can't tell - needs to be a wider control for confounders, no power calculation was conducted for the sample size, small proportion of women saw (but did not hold) the baby	Y	Y	Y<br><br>Highlights the need for more research on longer-term outcomes

Shapiro et al., 2017	Y	CT<br><br>Exact recruitment strategy unclear	Y	Y<br><br>Yes - although some measures were not explicitly named just described	Y<br><br>Controls for a wide range of confounders	Y	CT<br><br>Those lost to follow up were not reported	Y	Prior live term birth was associated with lower pregnancy anxiety in all three trimesters, whereas prior miscarriage was significantly associated with higher pregnancy anxiety in the first trimester<br><br>Prior stillbirth was associated with greater pregnancy anxiety in the third trimester	CIs moderate in regression analyses	Yes - large sample size, controls for a wide range of confounders but recruitment strategy could be described more	Y	Y	Y<br><br>Suggests further research on guilt

Treyvaud et al., 2016	Y	Y	Y	Y<br><br>Used validated scales for anxiety and depression	N<br><br>Only social risk was controlled for - it was not clear how this was decided or if other potential confounds were controlled for in the analysis	Y<br><br>Only social risk was controlled for - it was not clear how this was decided or if other potential confounds were controlled for in the analysis	Y<br><br>Those lost to follow up were not reported - just that there was some missing data but not clear if this was due to loss from follow up	Y	Compared with mothers who had not experienced bereavement, mothers who had were more likely to report elevated anxiety and depression symptoms at seven years (but not two years)	Confidence intervals are very large	Can’t tell – needs to be a wider control for confounders	Y	Y	Y<br><br>Highlights the need to be offered longer-term support as initial assessments may not reveal any difficulties

N/B. **Y=Yes, N=No, CT=Can’t tell. Comments are included in the same box where applicable. 6a & 6b greyed out where studies are cross-sectional.**

Supplementary Material (Table 6): **Critical appraisal for qualitative studies.**

	**Section A: Are the results valid?**						**Section B: What were the results?**			**Section C: Will the results help locally?**
	1. Was there a clear statement of the aims of the research? **HINT: What was the goal of the research? Why it was thought important? Its relevance**	2. Is a qualitative methodology (or quasi-experimental or mixed methods) appropriate? **HINT: Consider if the research seeks to interpret or illuminate the actions and/or subjective experiences of research participants. Is qualitative research the right methodology for addressing the research goal?**	3. Was the research design appropriate to address the aims of the research? **HINT: Consider if the researcher has justified the research design e.g., have they discussed how they decided which method to use**	4. Was the recruitment strategy appropriate to the aims of the research? **HINT: Consider if the researcher has explained how the participants were selected, if they explained why the participants they selected were the most appropriate to provide access to the type of knowledge sought after by the study, if there are any discussions around recruitment (e.g., why some people chose not to take part)**	5. Was the data collected in a way that addressed the research issue? **HINT: Consider if the setting for the data collection was justified, if it is clear how data were collected (e.g., focus group, semi-structured interview, etc.), if the researcher has justified the methods chosen, if the researcher has made the methods explicit (e.g., for interview method, is there an indication of how interviews are conducted, or did they use a topic guide), if methods were modified during the study if so has the researcher explained how and why, if the form of data is clear (e.g., tape recordings, video material, notes etc.), if the researcher has discussed**	6. Has the relationship between researcher and participants been adequately considered? **HINT: Consider if the researcher has critically examined their own role, potential bias and influence during (a) formulation of the research questions (b) data collection, including sample recruitment and choice of location. How has the researcher responded to events during the study and whether they considered the implications of any changes in the research design**	7. Have ethical issues been taken into consideration? **HINT: Consider if there are sufficient details of how the research was explained to participants for the reader to assess whether ethical standards were maintained, if the researcher has discussed issues raised by the study (e.g., issues around informed consent or confidentiality or how they have handled the effects of the study on the participants during and after the study, if approval has been sought from the ethics committee**	8. Was the data analysis sufficiently rigorous? **HINT: Consider if there is an in-depth description of the analysis process, if thematic analysis is used if so is it clear how the categories/themes were derived from the data, whether the researcher explains how the data presented were selected from the original sample to demonstrate the analysis process, if sufficient data are presented to support the findings, to what extent contradictory data are taken into account, whether the research critically examined their own role, potential bias and influence during analysis and selection of**	9. Is there a clear statement of findings? **HINT: Consider whether if the findings are explicit, if there is adequate discussion of the evidence both for and against the research arguments, if the researcher has discussed the credibility of their findings (e.g., triangulation, respondent validation, more than one analyst), if the findings are discussed in relation to the original research question**	10. How valuable is the research? **HINT: Consider if the researcher discusses the contribution the study makes to existing knowledge or understanding (e.g., do they consider the findings in relation to current practice or policy, or relevant research-based literature, if they identify new areas where research is necessary, if the researchers have discussed whether or how the findings can be transferred to other populations or considered other ways the research may be used**

					**saturation of data**			**data for presentation**		
Burkhammer et al., 2004	N	Y	Y	CT	CT	CT<br><br>Not mentioned	CT	CT	Y	Not discussed in detail
Kavanaugh & Robertson, 1999	Y	Y	Y	Y	Y	CT<br><br>Not mentioned	Y	CT	Y	Not discussed in detail
Shelkowitz et al., 2015	Y	Y<br><br>Quantitative was just descriptives of personal care options	CT<br><br>Method was not justified or discussed but is appropriate for the aims of the research	Y	Y	CT<br><br>Not mentioned	Y	CT	Y	Not discussed in detail

***N/B*. Y=Yes, N=No, CT=Can't Tell. Comments are included in the same box where applicable.**
